# Native American ancestry significantly contributes to neuromyelitis optica susceptibility in the admixed Mexican population

**DOI:** 10.1038/s41598-020-69224-3

**Published:** 2020-08-13

**Authors:** Sandra Romero-Hidalgo, José Flores-Rivera, Verónica Rivas-Alonso, Rodrigo Barquera, María Teresa Villarreal-Molina, Bárbara Antuna-Puente, Luis Rodrigo Macias-Kauffer, Marisela Villalobos-Comparán, Jair Ortiz-Maldonado, Neng Yu, Tatiana V. Lebedeva, Sharon M. Alosco, Juan Daniel García-Rodríguez, Carolina González-Torres, Sandra Rosas-Madrigal, Graciela Ordoñez, Jorge Luis Guerrero-Camacho, Irene Treviño-Frenk, Monica Escamilla-Tilch, Maricela García-Lechuga, Víctor Hugo Tovar-Méndez, Hanna Pacheco-Ubaldo, Victor Acuña-Alonzo, Maria-Cátira Bortolini, Carla Gallo, Gabriel Bedoya, Francisco Rothhammer, Rolando González-Jose, Andrés Ruiz-Linares, Samuel Canizales-Quinteros, Edmond Yunis, Julio Granados, Teresa Corona

**Affiliations:** 1 Departamento de Genómica Computacional, Instituto Nacional de Medicina Genómica (INMEGEN), 14610 Mexico City, Mexico; 2 Laboratorio Clínico de Enfermedades Neurodegenerativas, Instituto Nacional de Neurología y Neurocirugía “Manuel Velasco Suarez” (INNN), 14269 Mexico City, Mexico; 3 Molecular Genetics Laboratory, National School of Anthropology and History, 14030 Mexico City, Mexico; 4 Department of Archaeogenetics, Max Planck Institute for the Science of Human History, 07745 Jena, Germany; 5 Laboratorio de Enfermedades Cardiovasculares, INMEGEN, 14610 Mexico City, Mexico; 6 Unidad de Genómica de Poblaciones Aplicada a La Salud, Facultad de Química, UNAM/INMEGEN, 04510 Mexico City, Mexico; 7 HLA Laboratory, The American Red Cross Northeast Division, Dedham, MA 02026 USA; 8 Unidad de Secuenciación e Identificación de Polimorfismos, INMEGEN, 14610 Mexico City, Mexico; 9 Neuroimmunology, INNN, Mexico City, Mexico; 10 Neurogenetics Department, INNN, 14269 Mexico City, Mexico; 11 Department of Neurology, Instituto Nacional de Ciencias Medicas y Nutrición “Salvador Zubirán” (INCMNSZ), 14080 Mexico City, Mexico; 12 Neurologic Center, ABC Medical Center, Mexico City, Mexico; 13 Department of Transplantation, INCMNSZ, 14080 Mexico City, Mexico; 14 Departamento de Genética, Universidade Federal Do Rio Grande Do Sul, Porto Alegre, 91501-970 Brasil; 15 Laboratorios de Investigación y Desarrollo, Facultad de Ciencias y Filosofía, Universidad Peruana Cayetano Heredia, Lima 31, Peru; 16 GENMOL (Genetica Molecular), Universidad de Antioquia, 5001000 Medellin, Colombia; 17 Departamento de Tecnología Médica, Facultad de Ciencias de La Salud, Universidad de Tarapaca, 1000009 Arica, Chile; 18 Centro Nacional Patagónico, CONICET, Unidad de Diversidad, Sistematica Y Evolucion, Puerto Madryn U912OACD, Argentina; 19 Department of Genetics, Evolution and Environment, UCL Genetics Institute, University College London, London, WC1E 6BT UK; 20 Department of Cancer Immunology and Virology, Dana Farber Cancer Institute, Boston, MA 02215 USA

**Keywords:** Genetics, Immunology, Medical research, Risk factors

## Abstract

Neuromyelitis Optica (NMO) is an autoimmune disease with a higher prevalence in non-European populations. Because the Mexican population resulted from the admixture between mainly Native American and European populations, we used genome-wide microarray, HLA high-resolution typing and *AQP4* gene sequencing data to analyze genetic ancestry and to seek genetic variants conferring NMO susceptibility in admixed Mexican patients. A total of 164 Mexican NMO patients and 1,208 controls were included. On average, NMO patients had a higher proportion of Native American ancestry than controls (68.1% vs 58.6%; p = 5 × 10^–6^). GWAS identified a HLA region associated with NMO, led by rs9272219 (OR = 2.48, *P* = 8 × 10^–10^). Class II HLA alleles HLA-DQB1*03:01, -DRB1*08:02, -DRB1*16:02, -DRB1*14:06 and -DQB1*04:02 showed the most significant associations with NMO risk. Local ancestry estimates suggest that all the NMO-associated alleles within the HLA region are of Native American origin. No novel or missense variants in the *AQP4* gene were found in Mexican patients with NMO or multiple sclerosis. To our knowledge, this is the first study supporting the notion that Native American ancestry significantly contributes to NMO susceptibility in an admixed population, and is consistent with differences in NMO epidemiology in Mexico and Latin America.

## Introduction

Neuromyelitis optica (NMO) is a chronic autoimmune inflammatory and demyelinating disease of the central nervous system (CNS), which mainly affects the optic nerve and spinal cord. Although NMO was first described in the XIX century, it was considered a clinical variant of multiple sclerosis (MS) for decades^1,2^. In 2004, the discovery of positive antiaquaporin-4 antibodies (AQP4-IgG) in serum of the majority of NMO patients led to significant progress in the clinical characterization of the disease, now acknowledged as a distinct entity with different immunological, clinical and epidemiological features^3,4^.

Although it has been difficult to establish the actual prevalence of NMO, mainly because most reports are not comparable due to differences in study design, methodological approaches, and diagnostic criteria, worldwide NMO prevalence has been estimated between 0.51 and 4.4 cases per 100,000 inhabitants^6,7^. The prevalence and clinical manifestations of NMO vary among different ethnic groups, and several authors have stated that NMO is more frequent in non-European populations^8,9^. Interestingly, the relative frequency of NMO (estimated as the ratio of NMO/(MS + NMO) cases) has been found to decrease gradually in South America from North (Venezuela) to South (Argentina). Because ethnicity also changes gradually from North to South in this region, with the proportion of European individuals being lower in Venezuela and higher in Argentina, the authors suggested that ethnic origin influences NMO frequency in Latin America^10^. To date there are no population-based studies of the prevalence of NMO in Mexico, and there is a single study estimating NMO prevalence at 1.3 per 100,000 inhabitants based on the NMO/(MS + NMO) relative frequency at a referral center in Mexico City^12^.

Like many other autoimmune diseases, NMO is a multifactorial disorder that results from complex interactions between genetic and environmental factors. Recent studies have reported associations of NMO with genetic variation in the Human Leukocyte Antigen (HLA) genome region in chromosome 6, particularly with class II alleles, showing ethnical and geographical differences: The DRB1*03:01 allele has been associated with NMO in European^13–16^, Brazilian^17,18^, Afro-Caribbean^19^ and Mexican patients^20^; DRB1*16:02 in Southern Han Chinese^21^, Japanese^22^ and in Southern Brazilian patients^23^; DQB1*04:02 in a European-ancestry cohort^13,24^; and DRB1*04:05 in Southern Brazilians^23^. Moreover, candidate gene studies have reported associations with variation in non-HLA genes such as *AQP4* and others involved in immune function (*PD-1, IL-17, IL-7R, CD6* and *CD58*)^24–28^. Of the latter, only *AQP4* gene variation has been analyzed in various populations by sequencing promoter and/or coding regions of the gene attempting to identify variants involved in the pathogenesis of NMO. However, the association of *AQP4* gene variation with NMO remains unclear, with inconsistent findings among populations^29–34^.

Because the Mexican population of today resulted from a complex and ongoing admixture process involving mainly Native American and European genetic components, we hypothesized that Native American ancestry also contributes to NMO susceptibility in Mexican patients. We thus used genome-wide microarray data, HLA high-resolution typing and *AQP4* gene sequencing data to explore genetic ancestry and to seek genetic variants conferring susceptibility to NMO in Mexican patients.

## Results

The present study includes 164 patients with Neuromyelitis Optica (NMO) (79% female) and 1,208  controls (61% female).

### Population admixture analysis

**Figure 1:**
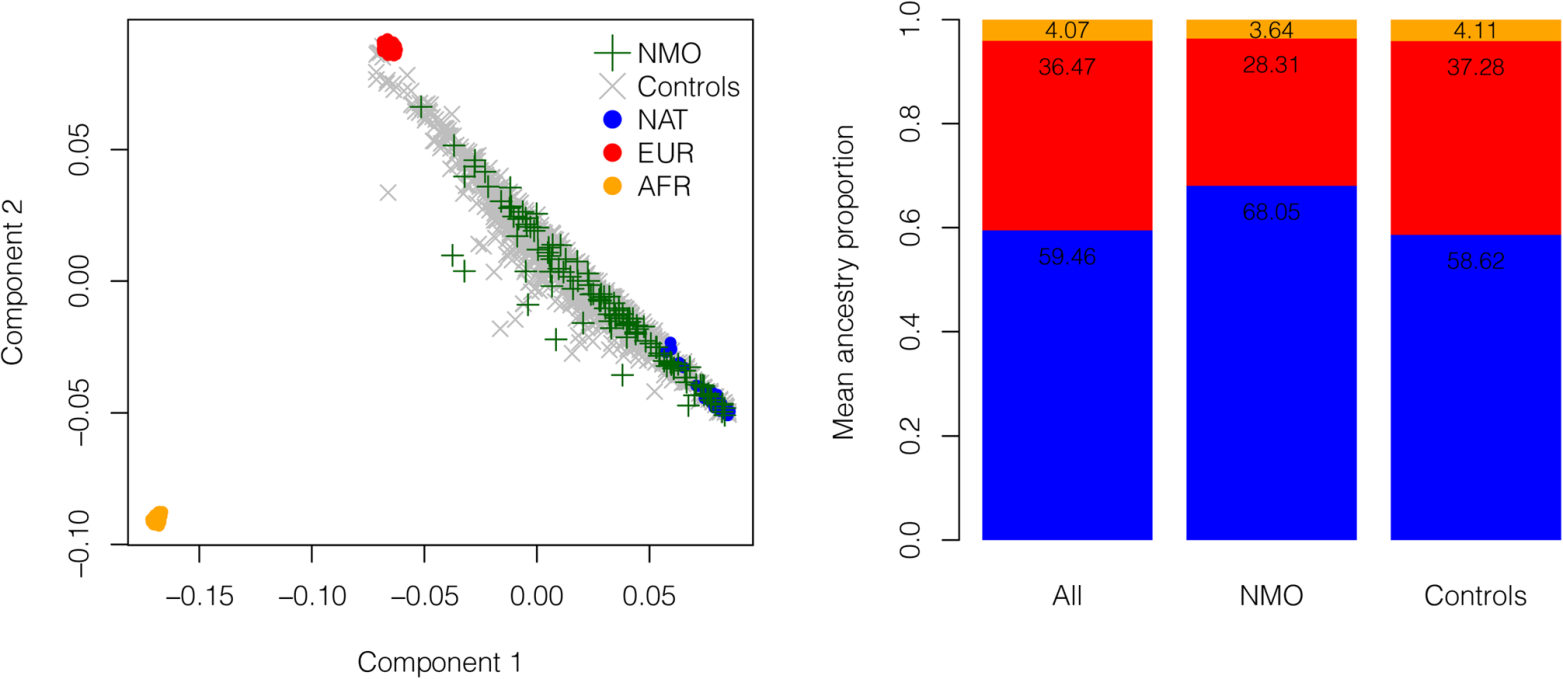
Global ancestry analysis. The left panel shows multidimensional scaling analysis of NMO patients and controls, with Europeans (CEU) and Africans (YRI) from the 1,000 Genomes projects, and unrelated central Native Mexican (NAT) individuals as reference populations. The right panel shows mean ancestry proportion estimated by ADMIXTURE assuming three parental populations (K = 3), in the entire study population, cases and controls.

Reference Native American (NAT) and continental populations were used to generate a multidimensional scaling (MDS) plot. Figure 1. The left panel shows that components 1 and 2 distinguish Africans (AFR) and Europeans (EUR) from NAT individuals. The average genome-wide proportion of Native American ancestry was significantly higher in NMO patients than in controls (68.1% *vs* 58.6%; *P* = 5 × 10^–6^). Conversely, the average genome-wide proportion of European ancestry was lower in cases than in controls (28.3% *vs* 37.3%; *P* = 3 × 10^–6^), while African ancestry was similar in both groups (3.6% *vs* 4.1%; *P* = 0.143) (Fig. 1, right panel).

### Genome-wide association analysis

**Figure 2:**
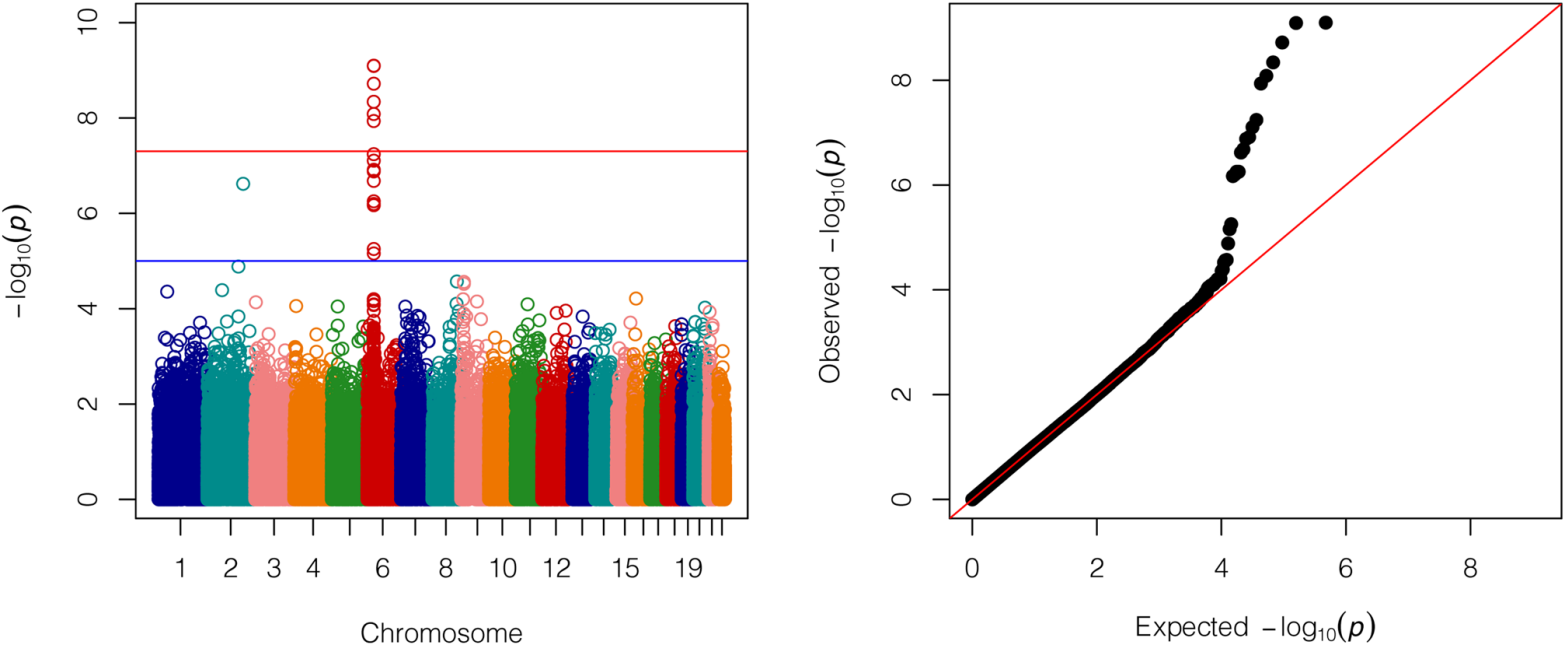
Genome-wide association analysis. Manhattan plot and Q–Q plot. *P*-values were obtained by logistic regression adjusting for sex and two principal components. Six signals surpassed the genome-wide significance threshold (5 × 10^–8^), spanning a 173.1 kb region in the major histocompatibility complex (MHC) region.

The genome-wide association analysis (GWAS) included 119 NMO cases and 1,208 controls. Figure 2 shows the Manhattan and Q-Q plots after adjusting for sex and two principal components. Six signals surpassed the genome-wide significance threshold (5 × 10^–8^), spanning a 173.1 kb region within the major histocompatibility complex (MHC) region. Two SNPs in perfect linkage disequilibrium (LD) (*r*^2^ = 1) showed the most significant associations under an additive model, rs9272219 (OR = 2.48, *P* = 8 × 10^–10^) and rs9273012 (OR = 2.49, *P* = 8 × 10^–10^), both within the HLA-DQA1 gene. As described in Table 1, two intergenic (rs1964995 and rs9271588) and two SNPs within the HLA-DRB9 gene (rs9368726 and rs9405108) were also significantly associated with NMO. LD estimations among these 6 SNPs ranged between 0.5 and 1 (0.5 < *r*^2^ < 1). In order to assess whether these associations are driven by a single SNP, we conditioned the analysis based on the rs9272219 genotype. No other associations maintained genome-wide significance in the conditioned analysis (Supplementary Fig. 1). Table 1 also shows associations of these SNPs with NMO using the data of Estrada et al. 2018^16^, available at https://github.com/Biogen-Inc/statgen (UTS/ACP data set).

**Table 1:** Association statistics for genome-wide significant SNPs.

rsID	Position	Gene	RA	RAF NMO (n = 119)	RAF Ctrl (n = 1,208)	Unconditioned		Conditioned on rs9272219		UTS/ACP data set	
						OR (95% CI)	*P*	OR (95% CI)	*P*	OR	*P*
rs9368726	32,438,542	HLA-DRB9	A	0.80	0.61	2.70 (1.92–3.70)	1 × 10^–8^	1.75 (1.09–2.86)	0.022	2.22	8 × 10^–9^
rs9405108	32,438,648	HLA-DRB9	G	0.80	0.61	2.70 (1.92–3.85)	8 × 10^–9^	1.75 (1.08–2.86)	0.023	2.22	8 × 10^–9^
rs1964995	32,449,411	Intergenic	A	0.78	0.56	2.63 (1.92–3.70)	5 × 10^–9^	1.79 (1.09–2.94)	0.023	–	–
rs9271588	32,590,953	Intergenic	A	0.75	0.53	2.63 (1.92–3.57)	2 × 10^–9^	1.75 (1.05–2.86)	0.032	1.96	1 × 10^–9^
rs9272219	32,602,269	HLA-DQA1	A	0.67	0.42	2.48 (1.86–3.32)	8 × 10^–10^	–	–	–	–
rs9273012	32,611,641	HLA-DQA1	G	0.67	0.42	2.49 (1.86–3.32)	8 × 10^–10^	–	–	–	–

RA, Risk allele. RAF: Risk allele frequency; UTS/ACP data set correspond to data obtained from Estrada et al. 2018^16^.

### HLA analysis

Detailed *HLA-A*, *HLA-B*, *HLA-C*, *HLA-DRB1*, and *HLA-DQB1* allele and haplotype frequencies in study groups are described in Supplementary Tables 1–9. No HLA variant deviated from Hardy–Weinberg equilibrium. Table 2 shows HLA alleles that differed significantly in frequency between NMO patients and controls. The most significantly NMO-associated HLA class II alleles (*P* < 0.01) were HLA-DQB1*03:01 (*P* = 0.00008), -DRB1*08:02 (*P* = 0.0011), -DRB1*16:02 (*P* = 0.0014), -DRB1*14:06 (*P* = 0.00368) and -DQB1*04:02 (*P* = 0.00552), all associated with increased NMO risk, while -DQB1*03:02 (*P* = 0.00013) and -DQB1*02:02 (*P* = 0.00899) were associated with decreased NMO risk. Two-locus HLA haplotypes associated with NMO patients are listed in Table 3. The lead class II haplotype associated with increased NMO risk was HLA-DRB1*16:02-DQB1*03:01 (*P* = 0.0014), followed by HLA-DRB1*08:02-DQB1*04:02 (*P* = 0.00177) and -DRB1*14:06-DQB1*03:01 (*P* = 0.00368), while -DRB1*07:01-DQB1*02:02 (*P* = 0.00796) was associated with decreased NMO risk. No extended haplotypes differed significantly between patients and controls.

**Table 2:** Comparison of HLA class I and class II allele frequencies in NMO patients and controls.

HLA Allele		NMO (n = 71)		Controls (n = 97)		*P*
		AF	n	AF	n	
Protective	B*39:05	0.0423	6	0.0979	19	0.04088
	DRB1*07:01	0.0282	4	0.0876	17	0.01996
	DRB1*04:07	0.0775	11	0.1598	31	0.01692
	DQB1*02:02	0.0282	4	0.0979	19	0.00899
	DQB1*03:02	0.1197	17	0.2887	56	0.00013
Risk	B*39:06	0.0775	11	0.0309	6	0.04818
	B*35:14	0.0493	7	0.0103	2	0.03275
	DQB1*04:02	0.2746	39	0.1546	30	0.00552
	DRB1*14:06	0.0915	13	0.0206	4	0.00368
	DRB1*16:02	0.1549	22	0.0515	10	0.00140
	DRB1*08:02	0.2676	38	0.1289	25	0.00110
	DQB1*03:01	0.3380	48	0.1546	30	0.00008

AF, Allele frequency. Only alleles with a frequency ≥ 0.01 in both groups and with significant differences between cases and controls are presented.

**Table 3:** Comparison of HLA class I and class II haplotype frequencies in NMO patients and controls.

HLA Haplotype		NMO (n = 71)			Controls (n = 97)			*P*
		HF	n	Δ′	HF	n	Δ′	
Protective	B*39:05-C*07:02	0.0423	6	1	0.0979	19	1	0.04088
	DRB1*04:07-DQB1*03:02	0.0704	10	0.8967	0.1546	30	1	0.01300
	DRB1*07:01-DQB1*02:02	0.0211	3	0.7428	0.0876	17	1	0.00796
Risk	B*35:14-C*04:01	0.0493	7	1	0.0103	2	1	0.03275
	B*39:06-C*07:02	0.0704	10	0.8805	0.0206	4	1	0.02422
	DRB1*14:06-DQB1*03:01	0.0915	13	1	0.0206	4	1	0.00368
	DRB1*08:02-DQB1*04:02	0.2606	37	0.9637	0.1289	25	1	0.00177
	DRB1*16:02-DQB1*03:01	0.1549	22	1	0.0515	10	1	0.00140

HF, Haplotype frequency. Only haplotypes with a frequency ≥ 0.01 in both groups and with significant differences between cases and controls are presented.

Table 4 shows HLA class II alleles stratified by rs9272219 alleles (C/A). Interestingly, previously reported risk alleles HLA-DQB1*04:02 and -DRB1*16:02, as well as HLA-DQB1*03:01, -DRB1*08:02 and -DRB1*14:06, all associated with NMO risk in the present study, were in strong linkage disequilibrium (LD) with the rs9272219 “A” risk allele. On the other hand, no NMO risk alleles, and two alleles associated with decreased NMO risk (-DQB1*03:02 and -DRB1*04:07) were in strong LD with the rs9272219 “C” allele.

**Table 4:** Comparison of *HLA-DRB1* and HLA*-DQB1* allele frequencies*,* stratified by rs9272219 genotype, in NMO patients and controls.

rs9272219	HLA Allele	NMO (n = 31)			Controls (n = 97)			P
		AF	n	Δ′	AF	n	Δ′	
C	DQB1*04:02	0.0323	2	1	0.0155	3	1	NS
	DRB1*04:04	0.0484	3	1	0.0619	12	0.8473	NS
	DRB1*04:07	0.0484	3	1	0.1598	31	0.94	0.01528
	DQB1*03:02	0.0968	6	1	0.2887	56	1	0.00107
	DQB1*03:03	0.0161	1	1	0.0103	2	1	NS
	DQB1*05:01	0.0323	2	1	0.0773	15	1	NS
	DQB1*06:01	0.0161	1	1	0.0155	3	0.6337	NS
	DQB1*06:02	0.0161	1	0.7338	0.0567	11	1	NS
	DQB1*06:04	0.0161	1	1	0.0309	6	1	NS
	DRB1*01:01	0.0161	1	1	0.0206	4	1	NS
	DRB1*01:02	0.0161	1	1	0.0258	5	1	NS
	DRB1*07:01	0.0161	1	1	0.0876	17	1	0.04056
	DRB1*13:02	0.0161	1	1	0.0361	7	1	NS
	DRB1*15:01	0.0161	1	0.7338	0.0567	11	1	NS
	DRB1*15:02	0.0161	1	1	0.0206	4	1	NS
A	DQB1*04:02	0.2742	17	1	0.1392	27	1	0.01415
	DRB1*03:01	0.0806	5	1	0.0412	8	1	NS
	DRB1*08:02	0.2903	18	1	0.1237	24	1	0.00279
	DRB1*14:02	0.0161	1	1	0.0309	6	1	NS
	DRB1*16:02	0.1613	10	1	0.0515	10	1	0.00831
	DQB1*03:01	0.3065	19	0.911	0.1546	30	1	0.00851
	DQB1*02:01	0.0806	5	0	0.0412	8	1	NS
	DQB1*03:19	0.0484	3	1	0.0103	2	1	NS
	DRB1*11:02	0.0323	2	1	0.0155	3	1	NS
	DRB1*11:04	0.0161	1	1	0.0206	4	1	NS
	DRB1*14:06	0.0968	6	0.7153	0.0206	4	1	0.01497

AF, allele frequency. Only Alleles with a frequency ≥ 0.01 in both groups are presented.

**Figure 3:**
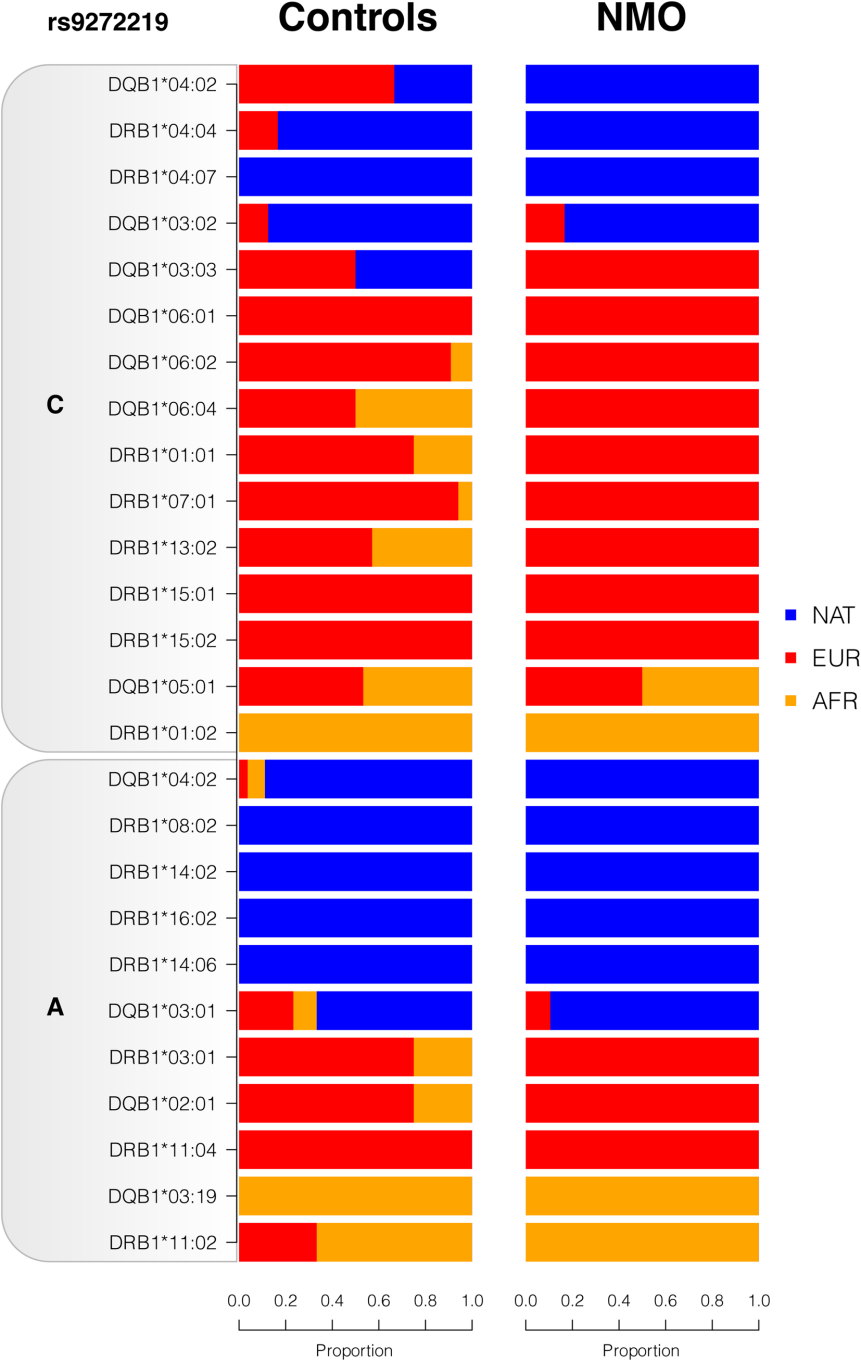
Local ancestry of HLA alleles. Proportion of *HLA* alleles described in Table 4 inferred as Native American (NAT), European (EUR) or African (AFR). Local ancestry was estimated using RFMIX using trio-phased populations as reference.

We then used RFMix to infer the local ancestry (NAT, EUR or AFR) of all alleles described in Table 4, in both cases and controls. Notably, all NMO risk alleles HLA-DRB1*08:02, -DRB1*16:02 and -DRB1*14:06 were inferred to be of Native American ancestry, while risk alleles -DQB1*04:02 and -DQB1*03:01 were predominantly of NAT ancestry both in cases and controls. Regarding protective NMO alleles, -DRB1*04:07 and -DQB1*03:02 alleles were also predominantly of NAT ancestry(Fig. 3).

### AQP4 sequencing

After sequencing all *AQP4* exons including exon–intron boundaries, we identified 35 SNPs in samples from NMO or MS Mexican patients. No novel variants were found. Thirteen of these variants were found in the 3′UTR region, one in the 5′UTR region, and three were synonymous variants. Although some variants were more frequent in NMO than in MS patients, the differences were not statistically significant. Supplementary Table 10, compares the alternative allele frequency of these 35 SNPs in NMO and MS patients, 1,000 Genomes continental populations and in 12 Native Mexican whole genome sequences^35^.

## Discussion

While several studies have stated that the epidemiology of NMO differs from that of MS, being more frequent in non-European populations^9–11^, reliable comparisons among studies are difficult to establish. Epidemiological data suggest that ethnicity influences NMO prevalence, particularly in Latin America^10^. In Mexico, there is only one non-population based study estimating NMO prevalence in a referral center located in Mexico City^12^. To our knowledge, this is the first study in the Mexican admixed population where a genome-wide analysis revealed a higher proportion of Native American ancestry in NMO cases as compared to controls. This contrasts with the higher European genetic component previously observed in the Mexican patients with MS^36^. The NAT ancestry estimated in our control group recruited in Mexico City (central Mexico) is consistent with previous NAT ancestry estimations in the Mexican Mestizo population (~ 55%), known to gradually decline from South to North throughout the Mexican territory^37,38^. Unfortunately, NMO epidemiological studies in Mexico are scarce, and there is no information on the geographical distribution or ethnicity of NMO patients in Mexico.

In our GWAS analysis, six SNPs within the MHC region were associated with NMO with genome-wide significance. Both linkage disequilibrium and the conditional association analyses suggest that this association is driven by a single signal, led by two SNPs in perfect linkage disequilibrium (rs9272219 and rs9273012). There is only one previous report of a GWAS for NMO in individuals of European ancestry^16^, where two independent signals in the same MHC region were significantly associated with NMO: rs28383224, which is 18.6 kb downstream and rs1150757, which is 573.1 kb downstream the lead SNP found in the present study (rs9272219). Although rs28383224 and rs1150757 genotypes were not available in our analysis, both NMO GWAS share data on 3 of the 6 SNPs associated with NMO in the Mexican cohort (rs9368726, rs9405108 and rs9271588). These 3 SNPs were also associated with increased NMO risk in the European cohort, although with slightly lower odds ratio values. It is important to point out that we found no SNPs in proximity of rs1150757 associated with NMO in the Mexican population.

Class II HLA alleles (HLA-DQB1*03:01, -DRB1*08:02, -DRB1*16:02, -DRB1*14:06 and -DQB1*04:02) and class II haplotypes (HLA-DRB1*16:02-DQB1*03:01, -DRB1*08:02-DQB1*04:02 and -DRB1*14:06-DQB1*03:01) showed the most significant associations with increased NMO risk in the present Mexican cohort, while HLA-DQB1*03:02 and -DQB1*02:02 alleles were significantly associated with decreased NMO risk. The HLA-DRB1*16:02 allele has also been associated with NMO in Southern Han Chinese and Japanese populations, and more recently in Southern Brazilians^21–23^. A very recent meta-analysis showed that the HLA-DRB1*16:02 allele was strongly associated with autoimmune diseases predominantly mediated by autoantibodies^5^. The frequency of this allele varies across the world but it is highest in Native populations of America (~ 39%), is also frequent in populations from Oceania (~ 28%) and South-East Asia (~ 28%), but is relatively low in Europe (~ 6%) and Africa (~ 4%)^39^. Furthermore, haplotype HLA-DRB1*16:02-DQB1*03:01 is very frequent in Native American populations from the Southern state of Oaxaca (Mixe, Mixtec and Zapotec) and Xavantes from Central Brazil, but is very rare in other continental populations^39–41^. Recently, haplotype HLA-DRB1*16-DQB1*03:01 was also associated with Parry-Romberg syndrome, an autoimmune disease affecting the craniofacial nerve in Mexican patients^42^.

The HLA-DRB1*03:01 allele has been consistently associated with NMO in European populations, and admixed populations with important contribution of the European gene pool (Brazilian mulatto, Afro-Caribbean and a small Mexican mestizo cohort)^13–20^. The frequency of HLA-DRB1*03:01 is as high as 20% in European, North African, Western Asian populations, but ranges from only 0 to 2% in Native Mexican populations^39^. Although in the present study the frequency of this allele was two-fold higher in cases as compared to controls (7.04% *vs* 4.12%, Supplementary Table 4), the difference did not reach statistical significance probably due to low statistical power derived from the small sample and effect sizes. Inconsistencies are not uncommon in genetic association studies and show the complexity of the genetic ancestry contribution in admixed populations. Interestingly, in the European GWAS, the HLA-DRB1*03:01 allele was imputed and found to be associated with AQP4-IgG-seropositive NMO but not with AQP4-IgG-seronegative NMO, and showed a high correlation with rs1150757 (r^2^ = 0.7) but a poor correlation with rs28383224 (r^2^ = 0.2)^16^. In the present study, the HLA-DRB1*03:01 allele was not associated with NMO, nor with any SNP in proximity of rs1150757.

HLA alleles previously associated with NMO in populations with European and/or Native American ancestry (HLA-DRB1*03:01^13–20^, -DRB1*16:02^21–23^ and -DQB1*04:02^13,24^) were in strong LD with the rs9272219 “A” risk allele. In contrast, two alleles significantly associated with decreased NMO risk in the present study (HLA-DRB1*04:07 and -DQB1*03:02) were in strong LD with the rs9272219 “C” allele. Whether the latter are in fact NMO protective alleles needs to be confirmed in independent cohorts. Moreover, no previously reported NMO risk alleles were found in individuals with the rs9272219 “C” allele.

Notably, local ancestry analyses revealed that all HLA alleles most associated with NMO risk and protection in the present study were predominantly inferred as of Native American ancestry. This is consistent with our finding of a higher proportion of NAT ancestry in NMO cases as compared to controls, and with epidemiological data suggesting that NMO is more prevalent in non-European populations^9–11^. To our knowledge there is only one previous study analyzing local ancestry of demyelinating diseases, where HLA alleles DRB1*16:02 and DRB1*14:02 were inferred as of Native American ancestry in Hispanics^43^, also in consistency with our local ancestry findings. As expected, the well-known HLA-DRB1*03:01 NMO risk allele was predominantly inferred as of European ancestry. Altogether, our SNP and HLA analyses suggest that a group of HLA alleles predominantly of Native American ancestry are associated with NMO susceptibility in the admixed Mexican population.

A limited number of studies have analyzed the role of *AQP4* variants in the pathogenesis of NMO in USA^29^, Chinese^30–32^, Japanese^33^ and Spanish^34^ populations, with inconclusive results. We sequenced *AQP4* coding regions in Mexican patients with NMO and MS, however no novel or missense variants were identified. Interestingly, four 3′UTR variants (rs7240333, rs14393, rs1058424 and rs3763043) were more frequent in NMO as compared to MS patients, although the differences were not significant. Two of these variants (rs1058424 and rs3763043) showed a weak but significant association with NMO in the Han Chinese population^31^. The highest frequencies of these four 3′UTR polymorphisms have been found in Native Mexicans (29.2%, 79.2%, 50% and 79.2%, respectively)^35^.

Some limitations of the study must be pointed out. Firstly, because no medical information was obtained from the control group (CANDELA project participants from Mexico), misclassification bias could potentially affect the statistical power of the study. Controls lacking medical information have been previously used in other GWAS studies, as the effect on statistical power is expected to be modest unless the extent of this bias is substantial^44^. In the present study, it is unlikely albeit possible, that a low number of control participants were affected with NMO or could eventually develop NMO in the future. However, because NMO prevalence is very low, this bias is expected to be small. In addition, because of the possibility of spurious associations, the novel HLA associations here identified should be interpreted with caution and be confirmed in further studies including Mexican and other Latin American populations.

To our knowledge, this is the first study to examine the genetic ancestry of NMO patients supporting the notion that Native American ancestry significantly contributes to Neuromyelitis optica susceptibility in the admixed Mexican population. This finding is consistent with differences in the prevalence of NMO in populations of Mexico and Latin America, and contrasts with the epidemiology and genetics of multiple sclerosis^36,45^.

## Methods

### Study population

A total of 164 Mexican NMO patients were included. NMO diagnosis was based on Wingerchuk criteria described in 2006 and revised in 2015^46,47^. All NMO participants had DNA samples stored at the National Institute of Neurology and Neurosurgery “Manuel Velasco Suárez” (INNN) DNA bank. Only DNA samples meeting quality requirements were used to perform microarray genotyping, high-resolution HLA typing and/or *AQP4* sequencing. From the 164 NMO patients, it was possible to perform microarray analysis in 119, high-resolution typing of the HLA region in 71 and *AQP4* sequencing in 48 cases. Only 25 cases were available for the three platforms and 31 have microarray and high-resolution HLA typing. Moreover*,* 1,208 individuals from the Consortium for the Analysis of the Diversity and Evolution of Latin America (CANDELA)^48^ were included as a control group for the genome-wide association analysis. *AQP4* sequencing data were compared in 48 NMO cases and 48 MS Mexican patients. All MS patients were recruited at the INNN and fulfilled the McDonald criteria for MS diagnosis^49^. A sample of 15 trios from central Native Mexican populations (Nahuas and Totonacs) were included as a Native American (NAT) reference panel for ancestry analyses. All experiments were performed in accordance with the relevant guidelines and regulations.

Informed consent was obtained from all participants, and the study was approved by the Ethics Committee of INNN for NMO and MS patients; Universidad Nacional Autónoma de México for CANDELA controls; and Instituto Nacional de Medicina Genómica (INMEGEN) for NAT trios.

### Genotyping

Eighty-three NMO samples were genotyped using the Illumina HumanOmniExpress array (~ 700,000 SNPs) and 36 using Illumina expanded Multi-Ethnic Genome Array (~ 1,700,000 SNPs), at the INMEGEN. Controls had been previously genotyped on *HumanOmniExpress* array as part of the CANDELA Consortium study^48^. One of the CANDELA-Mexico controls was also genotyped at INMEGEN for quality control purposes, and microarray data concordance was 99.8%.

### High-resolution typing of the HLA region

HLA class I (A, B and C) and class II (*DRB1* and *DQB1*) genes were typed by direct sequencing (sequence-based typing, SBT^50^) in a total of 71 NMO samples and 97 controls. Genotypes were called using *Applied Biosystems* analysis software (Foster City, CA, USA) and the *IMGT/HLA* database alignment tool^51^*.* Ambiguities were resolved using previously validated group-specific sequencing primers (GSSP)^50^.

### *AQP4* sequencing

*AQP4* coding and UTR regions were sequenced on a Illumina MiSeq system. Primers were designed manually to span the regions of interest. Quality control of raw sequences was conducted using FastQC^52^ and the Trimmomatic^53^ algorithm was used to remove adapter sequences and trim short and low-quality end-read sequences. By using Bowtie2^54^ and SAMTools^55^, we cleaned the sequence reads and then aligned them to the human reference genome (hg19) and variant calling and annotations.

### Statistical analysis

#### Genome-wide screening

Quality control (QC) of the genotype data was carried out in *PLINK*^56^. SNPs and individuals were removed from the analysis based on minor allele frequency < 5%, call rate < 95%, deviation from the Hardy–Weinberg equilibrium (HWE) at *P* < 1 × 10^–5^ and genotyping efficiency < 90%. Pairwise identity by descent (IBD) estimates were used to identify related individuals. No discordant sex information was found. After quality control, the data set comprised the genotypes of 119 NMO patients and 1,208 controls for 252,805 SNPs.

Ancestry analyses were carried out using *ADMIXTURE*^57^ assuming three parental populations: EUR and AFR from the 1,000 Genomes project, and NAT genotypes of unrelated individuals, i.e. only parents of the NAT trios were included. The total number of autosomal SNPs common to the five populations (NMO patients, CANDELA controls, EUR, AFR and NAT) was 197,323. For each individual, the proportion of European, African and Native American ancestry was estimated at the genomic level. The significance of differences between ancestry proportions in NMO patients as compared to controls was determined using a T-test statistic. Local ancestry was determined through a random forest procedure using RFMix with 5 expectation–maximization (EM) iterations and a minimum of 6 reference haplotypes per tree node^58^. EUR and AFR from 1,000 Genomes project, along with trio phased NAT genotypes were used as reference populations for local ancestry. Haplotype phasing of NAT, NMO and control subjects was performed with Beagle^59^.

A genome-wide case–control association study was conducted using logistic regression models, adjusting for sex and two principal components, assuming an additive effect using *PLINK*^56^.

#### HLA region analysis

Allele and haplotype frequencies were obtained by direct counting, and haplotype blocks were built based on previous reports. Allele frequencies for *HLA-A*, *-B*, *-C*, *-DRB1* and *-DQB1* were compared between NMO patients and the control group. Maximum-likelihood haplotype frequencies for two-point, three-point and four-point associations were estimated in each group using an EM algorithm implemented in Arlequin v3.1^60^. Linkage disequilibrium (LD; Δ and Δ′) and HWE were also calculated using Arlequin. Class II HLA haplotype were stratified by SNP rs9272219 and the significance of the differences between NMO patients and CANDELA controls was determined using a Fisher’s exact test.

## Supplementary information

Supplementary Information.

## Data Availability

The dataset for the NMO patients generated and/or analyzed during the current study are available from the corresponding authors on reasonable request. Access to the CANDELA dataset used in this manuscript was obtained through a formal request to the Consortium for the Analysis of the Diversity and Evolution of Latin America steering committee.
