# Supplementary material for: Native American ancestry significantly contributes to neuromyelitis optica susceptibility in the admixed Mexican population
Source: Sci Rep. 2020 Aug 13;10:13706. doi: 10.1038/s41598-020-69224-3 (PMC7426416; doi:10.1038/s41598-020-69224-3)
Supplement: Supplementary file 1 — Supplementary Information. [file 41598_2020_69224_MOESM1_ESM.docx]

**SUPPLEMENTARY MATERIAL**

**for**

**Native American ancestry significantly contributes to Neuromyelitis optica susceptibility in the admixed Mexican population**

Sandra Romero-Hidalgo^1*†^, José Flores-Rivera^2*^, Verónica Rivas-Alonso^2^, Rodrigo Barquera^3,4^, María Teresa Villarreal-Molina^5^, Bárbara Antuna-Puente^5^, Luis Rodrigo Macias-Kauffer^6^, Marisela Villalobos-Comparán^1^, Jair Ortiz-Maldonado^2^, Neng Yu^7^, Tatiana V. Lebedeva^7^, Sharon M. Alosco^7^, Juan Daniel García-Rodríguez^1^, Carolina González-Torres^8^, Sandra Rosas-Madrigal^5^, Graciela Ordoñez^9^, Jorge Luis Guerrero-Camacho^10^, Irene Treviño-Frenk^11,12^, Monica Escamilla-Tilch^13^, Maricela García-Lechuga^13^, Víctor Hugo Tovar-Méndez^13^, Hanna Pacheco-Ubaldo^3^, Victor Acuña-Alonzo^3^, Maria-Cátira Bortolini^14^, Carla Gallo^15^, Gabriel Bedoya^16^, Francisco Rothhammer^17^, Rolando González-Jose^18^, Andrés Ruiz-Linares^19^, Samuel Canizales-Quinteros^6^, Edmond Yunis^20^, Julio Granados^13†^, Teresa Corona^2†^

^1^Departamento de Genómica Computacional, Instituto Nacional de Medicina Genómica (INMEGEN), Mexico City 14610, Mexico.

^2^Laboratorio Clínico de Enfermedades Neurodegenerativas, Instituto Nacional de Neurología y Neurocirugía Manuel Velasco Suarez (INNN), Mexico City 14269, Mexico.

^3^Molecular Genetics Laboratory, National School of Anthropology and History, Mexico City 14030, Mexico.

^4^Department of Archaeogenetics, Max Planck Institute for the Science of Human History, Jena 07745, Germany.

^5^Laboratorio de Enfermedades Cardiovasculares, INMEGEN, Mexico City 14610, Mexico.

^6^Unidad de Genómica de Poblaciones Aplicada a la Salud, Facultad de Química, UNAM/INMEGEN, Mexico City 14610, Mexico.

^7^HLA Laboratory, The American Red Cross Northeast Division, Dedham, Massachusetts 02026, USA.

^8^ Unidad de Secuenciación e Identificación de Polimorfismos, INMEGEN, Mexico City 14610, Mexico.

^9^Neuroimmunology, INNN, Mexico City, Mexico.

^10^Neurogenetics Department, INNN, Mexico City 14269, Mexico.

^11^Department of Neurology, Instituto Nacional de Ciencias Medicas y Nutricion "Salvador Zubirán" (INCMNSZ), Mexico City 14080, Mexico.

^12^Neurologic Center, ABC Medical Center, Mexico City, Mexico

^13^Department of Transplantation, INCMNSZ, Mexico City 14080, Mexico.

^14^Departamento de Genética, Universidade Federal do Rio Grande do Sul, Porto Alegre 91501-970, Brasil.

^15^Laboratorios de Investigación y Desarrollo, Facultad de Ciencias y Filosofía, Universidad Peruana Cayetano Heredia, Lima 31, Peru.

^16^GENMOL (Genetica Molecular), Universidad de Antioquia, Medellin 5001000, Colombia.

^17^Departamento de Tecnología Médica, Facultad de Ciencias de la Salud, Universidad de Tarapaca, Arica 1000009, Chile.

^18^Centro Nacional Patagónico, CONICET, Unidad de Diversidad, Sistematica y Evolucion, Puerto Madryn U912OACD, Argentina.

^19^Department of Genetics, Evolution and Environment, UCL Genetics Institute, University College London, London WC1E 6BT, UK.

^20^Department of Cancer Immunology & Virology, Dana Farber Cancer Institute, MA 02215, USA.

*These authors contributed equally to this work.

^†^Correspondence and request for materials should be addressed to: S.R.-H. (email: sromero@inmege.gob.mx), T.C. (email: vcorona@unam.mx) and J.G. (email: julgrate@yahoo.com).

**A**

**B**

**Supplementary Figure 1.** Regional association plots. The x axis represents the chromosomal position, and the y axis is the –log10 of the P value of association. Colors indicate the LD level with the index SNP. A Regional association plot of the most significant signal represented by rs9272219. B Regional association plot conditioned the analysis based on the rs9272219 genotype. As observed, no associations maintained genome-wide significance in the conditional analysis.

**Supplementary Table 1.** Comparison of HLA-*A* allele frequencies in NMO cases and controls.

| **HLA-*A*** | | | | | |
| --- | --- | --- | --- | --- | --- |
| **Allele** | **NMO (n = 71)** | | **Controls (n = 97)** | | ***P*** |
|  | **AF** | **n** | **AF** | **n** |  |
| A*01:01 | 0.0493 | 7 | 0.0515 | 10 | NS |
| A*02:01 | 0.2254 | 32 | 0.2062 | 40 | NS |
| A*02:02 | 0.0070 | 1 | 0.0052 | 1 | NS |
| A*02:05 | 0.0352 | 5 | 0.0103 | 2 | NS |
| A*02:06 | 0.1056 | 15 | 0.0928 | 18 | NS |
| A*02:07 | 0.0000 | 0 | 0.0052 | 1 | NS |
| A*02:90 | 0.0070 | 1 | 0.0000 | 0 | NS |
| A*03:01 | 0.0141 | 2 | 0.0464 | 9 | NS |
| A*03:02 | 0.0070 | 1 | 0.0052 | 1 | NS |
| A*11:01 | 0.0352 | 5 | 0.0206 | 4 | NS |
| A*23:01 | 0.0282 | 4 | 0.0103 | 2 | NS |
| A*24:02 | 0.1972 | 28 | 0.1443 | 28 | NS |
| A*24:25 | 0.0000 | 0 | 0.0103 | 2 | NS |
| A*25:01 | 0.0070 | 1 | 0.0000 | 0 | NS |
| A*26:01 | 0.0141 | 2 | 0.0206 | 4 | NS |
| A*26:08 | 0.0000 | 0 | 0.0052 | 1 | NS |
| A*29:01 | 0.0141 | 2 | 0.0000 | 0 | NS |
| A*29:02 | 0.0282 | 4 | 0.0361 | 7 | NS |
| A*30:01 | 0.0000 | 0 | 0.0258 | 5 | NS |
| A*30:02 | 0.0070 | 1 | 0.0155 | 3 | NS |
| A*31:01 | 0.0563 | 8 | 0.0619 | 12 | NS |
| A*31:02 | 0.0000 | 0 | 0.0052 | 1 | NS |
| A*31:30 | 0.0070 | 1 | 0.0000 | 0 | NS |
| A*32:01 | 0.0000 | 0 | 0.0309 | 6 | 0.03584 |
| A*33:01 | 0.0282 | 4 | 0.0103 | 2 | NS |
| A*33:03 | 0.0000 | 0 | 0.0052 | 1 | NS |
| A*34:01 | 0.0000 | 0 | 0.0052 | 1 | NS |
| A*66:01 | 0.0000 | 0 | 0.0052 | 1 | NS |
| A*68:01 | 0.0634 | 9 | 0.0567 | 11 | NS |
| A*68:02 | 0.0282 | 4 | 0.0258 | 5 | NS |
| A*68:03 | 0.0211 | 3 | 0.0155 | 3 | NS |
| A*68:05 | 0.0141 | 2 | 0.0052 | 1 | NS |
| A*68:07 | 0.0000 | 0 | 0.0052 | 1 | NS |
| A*69:01 | 0.0000 | 0 | 0.0052 | 1 | NS |

AF: Allele frequency. NS: Not significant.

**Supplementary Table 2.** Comparison of HLA-*B* allele frequencies in NMO cases and controls.

| **HLA-*B*** | | | | | |
| --- | --- | --- | --- | --- | --- |
| **Allele** | **NMO (n = 71)** | | **Controls (n = 97)** | | ***P*** |
|  | **AF** | **n** | **AF** | **n** |  |
| B*07:02 | 0.0282 | 4 | 0.0515 | 10 | NS |
| B*07:05 | 0.0070 | 1 | 0.0000 | 0 | NS |
| B*07:15 | 0.0000 | 0 | 0.0052 | 1 | NS |
| B*08:01 | 0.0282 | 4 | 0.0361 | 7 | NS |
| B*08:02 | 0.0070 | 1 | 0.0000 | 0 | NS |
| B*13:02 | 0.0000 | 0 | 0.0155 | 3 | NS |
| B*14:01 | 0.0000 | 0 | 0.0103 | 2 | NS |
| B*14:02 | 0.0282 | 4 | 0.0258 | 5 | NS |
| B*15:01 | 0.0070 | 1 | 0.0206 | 4 | NS |
| B*15:03 | 0.0070 | 1 | 0.0052 | 1 | NS |
| B*15:07 | 0.0000 | 0 | 0.0052 | 1 | NS |
| B*15:15 | 0.0211 | 3 | 0.0052 | 1 | NS |
| B*15:16 | 0.0000 | 0 | 0.0052 | 1 | NS |
| B*15:17 | 0.0070 | 1 | 0.0103 | 2 | NS |
| B*15:21 | 0.0000 | 0 | 0.0052 | 1 | NS |
| B*15:30 | 0.0141 | 2 | 0.0103 | 2 | NS |
| B*15:35 | 0.0070 | 1 | 0.0000 | 0 | NS |
| B*15:39 | 0.0000 | 0 | 0.0052 | 1 | NS |
| B*15:48 | 0.0000 | 0 | 0.0052 | 1 | NS |
| B*18:01 | 0.0282 | 4 | 0.0258 | 5 | NS |
| B*27:05 | 0.0141 | 2 | 0.0309 | 6 | NS |
| B*35:01 | 0.0423 | 6 | 0.0309 | 6 | NS |
| B*35:02 | 0.0070 | 1 | 0.0206 | 4 | NS |
| B*35:03 | 0.0070 | 1 | 0.0103 | 2 | NS |
| B*35:12 | 0.0352 | 5 | 0.0464 | 9 | NS |
| B*35:14 | 0.0493 | 7 | 0.0103 | 2 | 0.03275 |
| B*35:16 | 0.0070 | 1 | 0.0052 | 1 | NS |
| B*35:17 | 0.0493 | 7 | 0.0258 | 5 | NS |
| B*35:20 | 0.0070 | 1 | 0.0000 | 0 | NS |
| B*35:24 | 0.0141 | 2 | 0.0000 | 0 | NS |
| B*35:43 | 0.0141 | 2 | 0.0052 | 1 | NS |
| B*37:01 | 0.0141 | 2 | 0.0052 | 1 | NS |
| B*38:01 | 0.0070 | 1 | 0.0103 | 2 | NS |
| B*39:01 | 0.0211 | 3 | 0.0052 | 1 | NS |
| B*39:02 | 0.0493 | 7 | 0.0052 | 1 | 0.01131 |
| B*39:05 | 0.0423 | 6 | 0.0979 | 19 | 0.04088 |
| B*39:06 | 0.0775 | 11 | 0.0309 | 6 | 0.04818 |
| B*39:08 | 0.0000 | 0 | 0.0103 | 2 | NS |
| B*39:10 | 0.0070 | 1 | 0.0000 | 0 | NS |
| B*39:24 | 0.0000 | 0 | 0.0052 | 1 | NS |
| B*40:01 | 0.0000 | 0 | 0.0052 | 1 | NS |
| B*40:02 | 0.0634 | 9 | 0.0361 | 7 | NS |
| B*40:04 | 0.0000 | 0 | 0.0052 | 1 | NS |
| B*40:05 | 0.0070 | 1 | 0.0206 | 4 | NS |
| B*40:08 | 0.0141 | 2 | 0.0000 | 0 | NS |
| B*40:11 | 0.0211 | 3 | 0.0052 | 1 | NS |
| B*40:20 | 0.0000 | 0 | 0.0052 | 1 | NS |
| B*40:27 | 0.0000 | 0 | 0.0103 | 2 | NS |
| B*41:01 | 0.0000 | 0 | 0.0052 | 1 | NS |
| B*41:02 | 0.0000 | 0 | 0.0052 | 1 | NS |
| B*44:02 | 0.0141 | 2 | 0.0258 | 5 | NS |
| B*44:03 | 0.0211 | 3 | 0.0361 | 7 | NS |
| B*45:01 | 0.0282 | 4 | 0.0103 | 2 | NS |
| B*46:01 | 0.0000 | 0 | 0.0052 | 1 | NS |
| B*47:01 | 0.0000 | 0 | 0.0052 | 1 | NS |
| B*48:01 | 0.0282 | 4 | 0.0206 | 4 | NS |
| B*48:03 | 0.0000 | 0 | 0.0052 | 1 | NS |
| B*49:01 | 0.0282 | 4 | 0.0155 | 3 | NS |
| B*50:01 | 0.0000 | 0 | 0.0052 | 1 | NS |
| B*50:02 | 0.0070 | 1 | 0.0000 | 0 | NS |
| B*51:01 | 0.0563 | 8 | 0.0515 | 10 | NS |
| B*51:02 | 0.0070 | 1 | 0.0000 | 0 | NS |
| B*52:01 | 0.0282 | 4 | 0.0361 | 7 | NS |
| B*53:01 | 0.0000 | 0 | 0.0309 | 6 | 0.03584 |
| B*55:01 | 0.0000 | 0 | 0.0052 | 1 | NS |
| B*57:01 | 0.0070 | 1 | 0.0000 | 0 | NS |
| B*57:03 | 0.0000 | 0 | 0.0052 | 1 | NS |
| B*58:01 | 0.0070 | 1 | 0.0000 | 0 | NS |
| B*58:02 | 0.0070 | 1 | 0.0000 | 0 | NS |

AF: Allele frequency. NS: Not significant.

**Supplementary Table 3.** Comparison of HLA-*C* allele frequencies in NMO cases and controls.

| **HLA-*C*** | | | | | |
| --- | --- | --- | --- | --- | --- |
| **Allele** | **NMO (n = 71)** | | **Controls (n = 97)** | | ***P*** |
|  | **AF** | **n** | **AF** | **n** |  |
| C*01:02 | 0.0563 | 8 | 0.0567 | 11 | NS |
| C*02:02 | 0.0282 | 4 | 0.0464 | 9 | NS |
| C*02:10 | 0.0070 | 1 | 0.0052 | 1 | NS |
| C*03:03 | 0.0211 | 3 | 0.0309 | 6 | NS |
| C*03:04 | 0.0634 | 9 | 0.0773 | 15 | NS |
| C*03:05 | 0.0141 | 2 | 0.0052 | 1 | NS |
| C*03:06 | 0.0282 | 4 | 0.0052 | 1 | NS |
| C*03:NEW | 0.0070 | 1 | 0.0000 | 0 | NS |
| C*04:01 | 0.2113 | 30 | 0.1546 | 30 | NS |
| C*04:03 | 0.0000 | 0 | 0.0052 | 1 | NS |
| C*05:01 | 0.0282 | 4 | 0.0412 | 8 | NS |
| C*05:09 | 0.0000 | 0 | 0.0052 | 1 | NS |
| C*06:02 | 0.0563 | 8 | 0.0464 | 9 | NS |
| C*07:01 | 0.0775 | 11 | 0.0567 | 11 | NS |
| C*07:02 | 0.2394 | 34 | 0.2165 | 42 | NS |
| C*07:04 | 0.0000 | 0 | 0.0052 | 1 | NS |
| C*08:01 | 0.0423 | 6 | 0.0309 | 6 | NS |
| C*08:02 | 0.0282 | 4 | 0.0309 | 6 | NS |
| C*12:02 | 0.0141 | 2 | 0.0155 | 3 | NS |
| C*12:03 | 0.0141 | 2 | 0.0258 | 5 | NS |
| C*14:02 | 0.0000 | 0 | 0.0103 | 2 | NS |
| C*14:03 | 0.0000 | 0 | 0.0052 | 1 | NS |
| C*15:02 | 0.0141 | 2 | 0.0258 | 5 | NS |
| C*15:05 | 0.0070 | 1 | 0.0052 | 1 | NS |
| C*15:09 | 0.0211 | 3 | 0.0103 | 2 | NS |
| C*16:01 | 0.0211 | 3 | 0.0309 | 6 | NS |
| C*17:01 | 0.0000 | 0 | 0.0052 | 1 | NS |
| C*18:01 | 0.0000 | 0 | 0.0052 | 1 | NS |

AF: Allele frequency. NS: Not significant.

**Supplementary Table 4.** Comparison of HLA-*DRB1* allele frequencies in NMO cases and controls.

| **HLA-*DRB1*** | | | | | |
| --- | --- | --- | --- | --- | --- |
| **Allele** | **NMO (n = 71)** | | **Controls (n = 97)** | | ***P*** |
|  | **AF** | **n** | **AF** | **n** |  |
| DRB1*01:01 | 0.0211 | 3 | 0.0206 | 4 | NS |
| DRB1*01:02 | 0.0141 | 2 | 0.0258 | 5 | NS |
| DRB1*01:03 | 0.0000 | 0 | 0.0103 | 2 | NS |
| DRB1*03:01 | 0.0704 | 10 | 0.0412 | 8 | NS |
| DRB1*03:02 | 0.0000 | 0 | 0.0052 | 1 | NS |
| DRB1*04:01 | 0.0070 | 1 | 0.0000 | 0 | NS |
| DRB1*04:02 | 0.0000 | 0 | 0.0103 | 2 | NS |
| DRB1*04:03 | 0.0070 | 1 | 0.0206 | 4 | NS |
| DRB1*04:04 | 0.0352 | 5 | 0.0619 | 12 | NS |
| DRB1*04:05 | 0.0000 | 0 | 0.0052 | 1 | NS |
| DRB1*04:06 | 0.0000 | 0 | 0.0052 | 1 | NS |
| DRB1*04:07 | 0.0775 | 11 | 0.1598 | 31 | 0.01692 |
| DRB1*04:11 | 0.0000 | 0 | 0.0361 | 7 | 0.02042 |
| DRB1*07:01 | 0.0282 | 4 | 0.0876 | 17 | 0.01996 |
| DRB1*08:01 | 0.0070 | 1 | 0.0103 | 2 | NS |
| DRB1*08:02 | 0.2676 | 38 | 0.1289 | 25 | 0.00110 |
| DRB1*08:03 | 0.0070 | 1 | 0.0000 | 0 | NS |
| DRB1*08:04 | 0.0070 | 1 | 0.0000 | 0 | NS |
| DRB1*09:01 | 0.0070 | 1 | 0.0155 | 3 | NS |
| DRB1*10:01 | 0.0070 | 1 | 0.0103 | 2 | NS |
| DRB1*11:01 | 0.0211 | 3 | 0.0052 | 1 | NS |
| DRB1*11:02 | 0.0141 | 2 | 0.0155 | 3 | NS |
| DRB1*11:03 | 0.0070 | 1 | 0.0000 | 0 | NS |
| DRB1*11:04 | 0.0070 | 1 | 0.0258 | 5 | NS |
| DRB1*12:01 | 0.0000 | 0 | 0.0103 | 2 | NS |
| DRB1*13:01 | 0.0000 | 0 | 0.0309 | 6 | 0.03584 |
| DRB1*13:02 | 0.0282 | 4 | 0.0361 | 7 | NS |
| DRB1*13:03 | 0.0000 | 0 | 0.0155 | 3 | NS |
| DRB1*13:05 | 0.0000 | 0 | 0.0052 | 1 | NS |
| DRB1*14:01 | 0.0000 | 0 | 0.0052 | 1 | NS |
| DRB1*14:02 | 0.0493 | 7 | 0.0309 | 6 | NS |
| DRB1*14:06 | 0.0915 | 13 | 0.0206 | 4 | 0.00368 |
| DRB1*15:01 | 0.0352 | 5 | 0.0567 | 11 | NS |
| DRB1*15:02 | 0.0282 | 4 | 0.0206 | 4 | NS |
| DRB1*15:03 | 0.0000 | 0 | 0.0052 | 1 | NS |
| DRB1*16:01 | 0.0000 | 0 | 0.0052 | 1 | NS |
| DRB1*16:02 | 0.1549 | 22 | 0.0515 | 10 | 0.00140 |
| DRB1*16:12 | 0.0000 | 0 | 0.0052 | 1 | NS |

AF: Allele frequency. NS: Not significant.

**Supplementary Table 5.** Comparison of HLA-*DQB1* allele frequencies in NMO cases and controls.

| **HLA-*DQB1*** | | | | | |
| --- | --- | --- | --- | --- | --- |
| **Allele** | **NMO (n = 71)** | | **Controls (n = 97)** | | ***P*** |
|  | **AF** | **n** | **AF** | **n** |  |
| DQB1*02:01 | 0.0704 | 10 | 0.0412 | 8 | NS |
| DQB1*02:02 | 0.0282 | 4 | 0.0979 | 19 | 0.00899 |
| DQB1*03:01 | 0.3380 | 48 | 0.1546 | 30 | 0.00008 |
| DQB1*03:02 | 0.1197 | 17 | 0.2887 | 56 | 0.00013 |
| DQB1*03:03 | 0.0070 | 1 | 0.0103 | 2 | NS |
| DQB1*03:19 | 0.0282 | 4 | 0.0103 | 2 | NS |
| DQB1*04:02 | 0.2746 | 39 | 0.1546 | 30 | 0.00552 |
| DQB1*04:03 | 0.0000 | 0 | 0.0052 | 1 | NS |
| DQB1*05:01 | 0.0423 | 6 | 0.0773 | 15 | NS |
| DQB1*05:02 | 0.0070 | 1 | 0.0103 | 2 | NS |
| DQB1*05:03 | 0.0000 | 0 | 0.0052 | 1 | NS |
| DQB1*06:01 | 0.0211 | 3 | 0.0155 | 3 | NS |
| DQB1*06:02 | 0.0352 | 5 | 0.0567 | 11 | NS |
| DQB1*06:03 | 0.0000 | 0 | 0.0412 | 8 | 0.01161 |
| DQB1*06:04 | 0.0282 | 4 | 0.0309 | 6 | NS |

AF: Allele frequency. NS: Not significant.

**Supplementary Table 6.** Comparison of HLA-*A-B* haplotype frequencies in NMO cases and controls.

| **HLA*-A-B*** | | | | | | | |
| --- | --- | --- | --- | --- | --- | --- | --- |
| **Haplotype** | **NMO (n = 71)** | | | **Controls (n = 97)** | | | ***P*** |
|  | **HF** | **n** | **Δ'** | **HF** | **n** | **Δ'** |  |
| A*01:01-B*08:01 | 0.0141 | 2 | 0.4741 | 0.0155 | 3 | 0.3957 | NS |
| A*01:01-B*52:01 | 0.0141 | 2 | 0.4741 | 0.0103 | 2 | 0.2447 | NS |
| A*02:01-B*35:01 | 0.0141 | 2 | 0.1394 | 0.0000 | 0 | ND | NS |
| A*02:01-B*35:12 | 0.0282 | 4 | 0.7418 | 0.0155 | 3 | 0.1481 | NS |
| A*02:01-B*35:14 | 0.0282 | 4 | 0.4468 | 0.0052 | 1 | 0.3611 | NS |
| A*02:01-B*35:17 | 0.0141 | 2 | 0.0779 | 0.0000 | 0 | ND | NS |
| A*02:01-B*39:06 | 0.0141 | 2 | -0.1932 | 0.0052 | 1 | 0.0417 | NS |
| A*02:05-B*37:01 | 0.0141 | 2 | 1.0000 | 0.0000 | 0 | ND | NS |
| A*02:06-B*15:15 | 0.0141 | 2 | 0.6273 | 0.0000 | 0 | ND | NS |
| A*02:06-B*39:02 | 0.0211 | 3 | 0.3611 | 0.0000 | 0 | ND | NS |
| A*02:06-B*39:05 | 0.0141 | 2 | 0.2546 | 0.0464 | 9 | 0.4424 | NS |
| A*02:06-B*39:06 | 0.0141 | 2 | 0.0852 | 0.0000 | 0 | ND | NS |
| A*02:06-B*48:01 | 0.0141 | 2 | 0.4409 | 0.0052 | 1 | 0.1687 | NS |
| A*11:01-B*51:01 | 0.0141 | 2 | 0.3642 | 0.0052 | 1 | 0.2069 | NS |
| A*23:01-B*18:01 | 0.0141 | 2 | 0.4855 | 0.0052 | 1 | 0.4860 | NS |
| A*24:02-B*07:02 | 0.0211 | 3 | 0.6886 | 0.0000 | 0 | ND | NS |
| A*24:02-B*35:17 | 0.0211 | 3 | 0.2882 | 0.0052 | 1 | 0.0564 | NS |
| A*24:02-B*39:01 | 0.0141 | 2 | 0.5848 | 0.0052 | 1 | 1.0000 | NS |
| A*24:02-B*39:06 | 0.0493 | 7 | 0.5471 | 0.0155 | 3 | 0.7051 | NS |
| A*24:02-B*40:02 | 0.0352 | 5 | 0.4464 | 0.0155 | 3 | 0.3260 | NS |
| A*24:02-B*51:01 | 0.0141 | 2 | 0.0658 | 0.0103 | 2 | 0.0564 | NS |
| A*29:02-B*44:03 | 0.0141 | 2 | 0.6570 | 0.0206 | 4 | 0.5545 | NS |
| A*33:01-B*14:02 | 0.0141 | 2 | 0.4855 | 0.0103 | 2 | 1.0000 | NS |
| A*68:01-B*14:02 | 0.0141 | 2 | 0.4662 | 0.0000 | 0 | ND | NS |
| A*68:01-B*39:02 | 0.0141 | 2 | 0.2374 | 0.0000 | 0 | ND | NS |
| A*68:01-B*40:08 | 0.0141 | 2 | 1.0000 | 0.0000 | 0 | ND | NS |
| A*68:02-B*49:01 | 0.0141 | 2 | 0.4855 | 0.0000 | 0 | ND | NS |
| A*68:03-B*39:05 | 0.0141 | 2 | 0.6520 | 0.0103 | 2 | 0.6283 | NS |

HF: Haplotype frequency. NS: Not significant. ND: Not determined. Only haplotypes with a frequency ≥0.01 in NMO are presented.

**Supplementary Table 7.** Comparison of HLA-*B-C* haplotype frequencies in NMO cases and controls.

| **HLA*-B-C*** | | | | | | | |
| --- | --- | --- | --- | --- | --- | --- | --- |
| **Haplotype** | **NMO (n = 71)** | | | **Controls (n = 97)** | | | ***P*** |
|  | **HF** | **n** | **Δ'** | **HF** | **n** | **Δ'** |  |
| B*07:02-C*07:02 | 0.0282 | 4 | 1.0000 | 0.0464 | 9 | 0.8704 | NS |
| B*08:01-C*07:01 | 0.0211 | 3 | 0.7290 | 0.0258 | 5 | 0.6961 | NS |
| B*14:02-C*08:02 | 0.0282 | 4 | 1.0000 | 0.0206 | 4 | 0.7933 | NS |
| B*15:15-C*01:02 | 0.0211 | 3 | 1.0000 | 0.0052 | 1 | 1.0000 | NS |
| B*18:01-C*05:01 | 0.0211 | 3 | 0.7428 | 0.0103 | 2 | 0.3798 | NS |
| B*27:05-C*02:02 | 0.0141 | 2 | 1.0000 | 0.0258 | 5 | 0.8248 | NS |
| B*35:12-C*04:01 | 0.0352 | 5 | 1.0000 | 0.0464 | 9 | 1.0000 | NS |
| B*35:14-C*04:01 | 0.0493 | 7 | 1.0000 | 0.0103 | 2 | 1.0000 | 0.0328 |
| B*35:17-C*03:04 | 0.0141 | 2 | 0.2374 | 0.0052 | 1 | 0.1290 | NS |
| B*35:17-C*04:01 | 0.0282 | 4 | 0.4566 | 0.0206 | 4 | 0.7610 | NS |
| B*35:24-C*04:01 | 0.0141 | 2 | 1.0000 | 0.0000 | 0 | ND | NS |
| B*35:43-C*01:02 | 0.0141 | 2 | 1.0000 | 0.0052 | 1 | 1.0000 | NS |
| B*37:01-C*06:02 | 0.0141 | 2 | 1.0000 | 0.0052 | 1 | 1.0000 | NS |
| B*39:01-C*07:02 | 0.0211 | 3 | 1.0000 | 0.0052 | 1 | 1.0000 | NS |
| B*39:02-C*07:02 | 0.0423 | 6 | 0.8122 | 0.0052 | 1 | 1.0000 | 0.0242 |
| B*39:05-C*07:02 | 0.0423 | 6 | 1.0000 | 0.0979 | 19 | 1.0000 | 0.0409 |
| B*39:06-C*07:02 | 0.0704 | 10 | 0.8805 | 0.0206 | 4 | 1.0000 | 0.0242 |
| B*40:02-C*03:04 | 0.0141 | 2 | 0.1696 | 0.0155 | 3 | 0.3779 | NS |
| B*40:02-C*03:06 | 0.0211 | 3 | 0.7331 | 0.0052 | 1 | 1.0000 | NS |
| B*40:02-C*04:01 | 0.0141 | 2 | 0.0139 | 0.0000 | 0 | ND | NS |
| B*40:11-C*03:04 | 0.0141 | 2 | 0.6441 | 0.0052 | 1 | 1.0000 | NS |
| B*44:03-C*16:01 | 0.0141 | 2 | 0.6595 | 0.0258 | 5 | 0.8267 | NS |
| B*45:01-C*06:02 | 0.0211 | 3 | 0.7351 | 0.0052 | 1 | 0.4743 | NS |
| B*48:01-C*08:01 | 0.0282 | 4 | 1.0000 | 0.0206 | 4 | 1.0000 | NS |
| B*49:01-C*07:01 | 0.0282 | 4 | 1.0000 | 0.0155 | 3 | 1.0000 | NS |
| B*51:01-C*15:02 | 0.0141 | 2 | 1.0000 | 0.0206 | 4 | 0.7885 | NS |
| B*51:01-C*15:09 | 0.0141 | 2 | 0.6468 | 0.0052 | 1 | 0.4713 | NS |
| B*52:01-C*03:03 | 0.0141 | 2 | 0.6570 | 0.0206 | 4 | 0.6535 | NS |
| B*52:01-C*12:02 | 0.0141 | 2 | 1.0000 | 0.0155 | 3 | 1.0000 | NS |

HF: Haplotype frequency. NS: Not significant. ND: Not determined. Only haplotypes with a frequency ≥0.01 in NMO are presented.

**Supplementary Table 8.** Comparison of HLA-*DRB1-DQB1* haplotype frequencies in NMO cases and controls.

| **HLA*-DRB1-DQB1*** | | | | | | | |
| --- | --- | --- | --- | --- | --- | --- | --- |
| **Haplotype** | **NMO (n = 71)** | | | **Controls (n = 97)** | | | ***P*** |
|  | **HF** | **n** | **Δ'** | **HF** | **n** | **Δ'** |  |
| DRB1*01:01-DQB1*05:01 | 0.0211 | 3 | 1.0000 | 0.0206 | 4 | 1.0000 | NS |
| DRB1*01:02-DQB1*05:01 | 0.0141 | 2 | 1.0000 | 0.0258 | 5 | 1.0000 | NS |
| DRB1*03:01-DQB1*02:01 | 0.0704 | 10 | 1.0000 | 0.0412 | 8 | 1.0000 | NS |
| DRB1*04:04-DQB1*03:02 | 0.0282 | 4 | 0.7728 | 0.0619 | 12 | 1.0000 | NS |
| DRB1*04:07-DQB1*03:02 | 0.0704 | 10 | 0.8967 | 0.1546 | 30 | 1.0000 | 0.0130 |
| DRB1*07:01-DQB1*02:02 | 0.0211 | 3 | 0.7428 | 0.0876 | 17 | 1.0000 | 0.0080 |
| DRB1*08:02-DQB1*04:02 | 0.2606 | 37 | 0.9637 | 0.1289 | 25 | 1.0000 | 0.0018 |
| DRB1*11:01-DQB1*03:01 | 0.0141 | 2 | 0.4965 | 0.0052 | 1 | 1.0000 | NS |
| DRB1*11:02-DQB1*03:19 | 0.0141 | 2 | 1.0000 | 0.0103 | 2 | 1.0000 | NS |
| DRB1*13:02-DQB1*06:04 | 0.0282 | 4 | 1.0000 | 0.0309 | 6 | 1.0000 | NS |
| DRB1*14:02-DQB1*03:01 | 0.0423 | 6 | 0.7842 | 0.0309 | 6 | 1.0000 | NS |
| DRB1*14:06-DQB1*03:01 | 0.0915 | 13 | 1.0000 | 0.0206 | 4 | 1.0000 | 0.0037 |
| DRB1*15:01-DQB1*06:02 | 0.0352 | 5 | 1.0000 | 0.0515 | 10 | 0.9033 | NS |
| DRB1*15:02-DQB1*06:01 | 0.0211 | 3 | 1.0000 | 0.0155 | 3 | 1.0000 | NS |
| DRB1*16:02-DQB1*03:01 | 0.1549 | 22 | 1.0000 | 0.0515 | 10 | 1.0000 | 0.0014 |

HF: Haplotype frequency. NS: Not significant. ND: Not determined. Only haplotypes with a frequency ≥0.01 in NMO are presented.

**Supplementary Table 9.** Comparison of HLA-*A-B-DRB1-DQB1* haplotype frequencies in NMO cases and controls.

| **HLA*-A-B-C-DRB1-DQB1*** | | | | | |
| --- | --- | --- | --- | --- | --- |
| **Haplotype** | **NMO (n = 71)** | | **Controls (n = 97)** | | ***P*** |
|  | **HF** | **n** | **HF** | **n** |  |
| A*24:02-B*39:06-C*07:02-DRB1*14:06-DQB1*03:01 | 0.0282 | 4 | 0.0052 | 1 | NS |
| A*02:01-B*35:12-C*04:01-DRB1*08:02-DQB1*04:02 | 0.0211 | 3 | 0.0103 | 2 | NS |
| A*02:06-B*39:02-C*07:02-DRB1*16:02-DQB1*03:01 | 0.0141 | 2 | 0.0000 | 0 | NS |
| A*02:01-B*35:14-C*04:01-DRB1*16:02-DQB1*03:01 | 0.0141 | 2 | 0.0052 | 1 | NS |
| A*68:01-B*39:02-C*07:02-DRB1*16:02-DQB1*03:01 | 0.0141 | 2 | 0.0000 | 0 | NS |
| A*68:01-B*14:02-C*08:02-DRB1*01:02-DQB1*05:01 | 0.0141 | 2 | 0.0000 | 0 | NS |
| A*23:01-B*18:01-C*05:01-DRB1*11:02-DQB1*03:19 | 0.0141 | 2 | 0.0000 | 0 | NS |
| A*01:01-B*52:01-C*12:02-DRB1*15:02-DQB1*06:01 | 0.0141 | 2 | 0.0103 | 2 | NS |
| A*02:06-B*39:05-C*07:02-DRB1*04:07-DQB1*03:02 | 0.0141 | 2 | 0.0309 | 6 | NS |
| A*01:01-B*08:01-C*07:01-DRB1*03:01-DQB1*02:01 | 0.0141 | 2 | 0.0103 | 2 | NS |
| A*02:06-B*15:15-C*01:02-DRB1*08:02-DQB1*04:02 | 0.0141 | 2 | 0.0000 | 0 | NS |
| A*02:05-B*37:01-C*06:02-DRB1*08:02-DQB1*04:02 | 0.0141 | 2 | 0.0000 | 0 | NS |
| A*68:02-B*49:01-C*07:01-DRB1*15:01-DQB1*06:02 | 0.0141 | 2 | 0.0000 | 0 | NS |

HF: Haplotype frequency. NS: Not significant. ND: Not determined. Only haplotypes with a frequency ≥0.01 in NMO are presented.

**Supplementary Table 10.** Frequency of 35 SNPs identified in NMO and/or multiple sclerosis (MS) patients.

| **Chr** | **Position** | **rsID** | **Type** | **Ref/Alt** | **NMO** | **MS** | **12G** | **AMR** | **EUR** | **AFR** | **EAS** | **SAS** |
| --- | --- | --- | --- | --- | --- | --- | --- | --- | --- | --- | --- | --- |
| 18 | 24432126 | rs757796172 | UTR3 | G/A | 0.0% | 4.2% |  | 0.0% | 0.0% | 0.0% | 0.0% | 0.0% |
| 18 | 24432276 | rs527336734 | UTR3 | A/G | 0.0% | 1.0% |  | 1.7% | 0.0% | 0.2% | 0.0% | 0.0% |
| 18 | 24432607 | rs117140070 | UTR3 | C/G | 0.0% | 2.1% |  | 0.7% | 1.6% | 0.8% | 0.0% | 0.3% |
| 18 | 24432812 | rs7240333 | UTR3 | C/T | 17.7% | 12.5% | 29.2% | 13.0% | 10.0% | 2.2% | 1.4% | 5.6% |
| 18 | 24432909 | rs117796128 | UTR3 | C/G | 0.0% | 1.0% |  | 0.7% | 0.8% | 0.0% | 0.0% | 0.3% |
| 18 | 24434263 | rs190220032 | UTR3 | A/C | 1.0% | 0.0% |  | 0.0% | 0.0% | 0.1% | 0.0% | 0.0% |
| 18 | 24434530 | rs73945976 | UTR3 | C/G | 2.1% | 2.1% |  | 2.2% | 2.2% | 6.0% | 0.4% | 11.0% |
| 18 | 24435095 | rs1058427 | UTR3 | G/T | 9.4% | 10.4% |  | 10.0% | 12.0% | 0.7% | 0.1% | 1.8% |
| 18 | 24435212 | rs14393 | UTR3 | G/T | 52.1% | 37.5% | 79.2% | 49.0% | 30.0% | 14.0% | 40.0% | 25.0% |
| 18 | 24435545 | rs1058424 | UTR3 | A/T | 37.5% | 25.0% | 50.0% | 36.0% | 20.0% | 7.7% | 41.0% | 19.0% |
| 18 | 24435587 | rs335929 | UTR3 | A/C | 11.5% | 13.5% | 8.3% | 11.0% | 19.0% | 1.4% | 39.0% | 34.0% |
| 18 | 24435818 | rs3763043 | UTR3 | C/T | 52.1% | 39.6% | 79.2% | 50.0% | 31.0% | 22.0% | 40.0% | 25.0% |
| 18 | 24441215 | rs1839318 | synonymous | C/T | 1.0% | 2.1% |  | 1.7% | 2.2% | 4.4% | 0.4% | 11.0% |
| 18 | 24441479 | rs113455094 | intronic | C/T | 2.1% | 0.0% |  | 0.0% | 0.0% | 0.1% | 0.1% | 0.0% |
| 18 | 24441657 | rs73945981 | intronic | G/T | 1.0% | 0.0% |  | 0.3% | 0.0% | 5.1% | 0.0% | 0.0% |
| 18 | 24441661 | rs74163677 | intronic | G/A | 2.1% | 2.1% |  | 1.7% | 4.6% | 0.2% | 7.2% | 4.3% |
| 18 | 24441786 | rs55875625 | intronic | T/C | 9.4% | 10.4% |  | 10.0% | 12.0% | 0.7% | 0.2% | 1.8% |
| 18 | 24442056 | rs455671 | intronic | A/G | 11.5% | 13.5% | 8.3% | 11.0% | 19.0% | 3.4% | 39.0% | 34.0% |
| 18 | 24442227 | rs72557968 | synonymous | C/T | 1.0% | 2.1% |  | 1.7% | 2.2% | 1.1% | 0.4% | 11.0% |
| 18 | 24442392 | rs35248760 | synonymous | C/A | 9.4% | 10.4% |  | 10.0% | 12.0% | 0.7% | 0.1% | 1.8% |
| 18 | 24442967 | rs8086336 | intronic | G/C | 0.0% | 2.1% |  | 2.2% | 1.1% | 8.3% | 0.0% | 0.2% |
| 18 | 24443028 | rs112481323 | intronic | C/G | 0.0% | 2.1% |  | 3.3% | 1.1% | 24.0% | 0.0% | 0.2% |
| 18 | 24443052 | rs72878776 | intronic | G/A | 9.4% | 9.4% |  | 10.0% | 12.0% | 0.7% | 0.1% | 1.8% |
| 18 | 24443075 | rs77905661 | intronic | G/A | 1.0% | 2.1% |  | 1.9% | 3.9% | 3.6% | 0.4% | 12.0% |
| 18 | 24443421 | rs63514 | intronic | C/T | 9.4% | 10.4% | 8.3% | 11.0% | 19.0% | 12.0% | 38.0% | 29.0% |
| 18 | 24444150 | rs12968026 | intronic | T/C | 9.4% | 9.4% |  | 10.0% | 12.0% | 2.9% | 0.1% | 1.8% |
| 18 | 24444214 | rs162009 | intronic | G/A | 19.8% | 21.9% | 8.3% | 24.0% | 33.0% | 34.0% | 36.0% | 31.0% |
| 18 | 24444374 | rs3763040* | intronic | G/A | 17.7% | 25.0% | 29.2% | 17.3% | 20.0% | 6.1% | 13.9% | NA |
| 18 | 24444449 | rs59439491 | intronic | G/C | 0.0% | 2.1% |  | 2.9% | 1.1% | 12.0% | 0.0% | 0.1% |
| 18 | 24444486 | rs59417158 | intronic | G/A | 1.0% | 0.0% |  | 0.4% | 0.0% | 6.2% | 0.0% | 0.0% |
| 18 | 24444981 | rs4800773 | intronic | G/A | 28.1% | 38.5% | 29.2% | 34.0% | 37.0% | 44.0% | 16.0% | 24.0% |
| 18 | 24445375 | rs149811972 | intronic | G/A | 12.5% | 10.4% | 12.5% | 3.9% | 0.2% | 0.1% | 0.0% | 0.1% |
| 18 | 24445433 | rs3875089 | intronic | T/C | 11.5% | 11.5% |  | 14.0% | 16.0% | 32.0% | 0.5% | 14.0% |
| 18 | 24445514 | rs111416077 | intronic | T/G | 0.0% | 2.1% |  | 2.0% | 1.1% | 2.4% | 0.0% | 0.1% |
| 18 | 24445692 | rs162008 | UTR5 | C/T | 10.4% | 10.4% | 8.3% | 10.0% | 20.0% | 8.9% | 36.0% | 29.0% |
| *Frequency obtained from Genome Aggregation Database (gnomAD); NA, not available frequency in gnomAD; AMR, EUR, AFR, EAS, SAS correspond to 1000 Genome Project continental populations and 12G corresponds to 12 Native Mexican whole genome sequences^1^. | | | | | | | | | | | | |

1 Romero-Hidalgo, S. *et al.* Demographic history and biologically relevant genetic variation of Native Mexicans inferred from whole-genome sequencing. *Nat Commun* **8**, 1005, doi:10.1038/s41467-017-01194-z (2017).
